# Tetracycline residue alters the nutritional quality and bioactive composition of soybean sprouts: Evidence from transcriptomic and rhizosphere microbiota analyses

**DOI:** 10.1016/j.fochms.2025.100345

**Published:** 2025-12-31

**Authors:** Ting Cai, Jie Yao, Hongmei Jiang, Jie Zou, Ting Xia, Xinyue Mou, Shan Zhang, Xiao Tan, Jie Tang, Wenliang Xiang

**Affiliations:** a School of Food and Bioengineering, Xihua University, Chengdu 610039, Sichuan, China; b Food Microbiology Key Laboratory of Sichuan Province, Xihua University, Chengdu 610039, Sichuan, China; c College of Chemistry and Life Science, Chengdu Normal University, Chengdu 611130, Sichuan, China

**Keywords:** Antibiotic contamination, Agricultural systems, Edible crops, Nutritional quality, Nitrogen-cycling microorganisms

## Abstract

Antibiotic residues in edible crops have become an increasing food safety concern, yet their impacts on crop nutritional quality and bioactive composition remain poorly understood. Here, we investigated the effects of tetracycline, a widely used antibiotic in soil–vegetable systems, on the growth and nutritional quality and bioactive composition of soybean sprouts. Results showed tetracycline exposure significantly inhibited sprout growth and nutrient accumulation in a dose-dependent manner, with high concentrations reducing vitamin C, total flavonoids, and coumestrol contents by approximately 50 %, 30 %, and 43 %, respectively. Transcriptomic analysis revealed that these related declines were associated with disruptions in carbon, amino acid, and lipid metabolism, as well as in flavonoid and coumestrol biosynthesis pathways. In parallel, rhizosphere microbiota analysis showed that tetracycline reshaped microbial community structure by reducing nitrogen-cycling-related taxa (*Dokdonella*, *Acidibacter*) and enriching resistant genera (*Acinetobacter)*, which were significantly correlated with changes in sprout nutritional quality and bioactive composition. Together, these results demonstrate that tetracycline residues drive substantial losses of nutritional and bioactive composition in edible crops through coordinated metabolic and microbiome-mediated mechanisms, revealing an underappreciated pathway by which antibiotic contamination threatens crop nutritional value and food quality.

## Highlights

• Tetracycline residue suppressed soybean sprout growth and nutrient quality.
• Vitamin C, total flavonoids, and coumestrol decreased by approximately 50 %, 30 %, and 43 %, respectively.
• Tetracycline interfered with core metabolic pathways in soybean sprouts.
• Tetracycline altered rhizosphere microbes involved in nitrogen cycling.
• Microbial shifts under tetracycline were linked to nutrient loss and stunting.

## 1 Introduction

Antibiotics are widely recognized as environmental contaminants, and increasing attention has been paid to the potential safety risks associated with their residues in edible crops (Yin et al., 2023). Due to their extensive use in aquaculture, livestock production, and medical applications, multiple antibiotic classes (e.g., tetracyclines, β-lactams, and aminoglycosides) are continuously introduced into agroecosystems and are frequently detected in agricultural soils, irrigation water, and organic fertilizers (Russell et al., 2025; Wang et al., 2024). Among the various exposure pathways, soil–vegetable systems represent a primary route through which residual antibiotics in agroecosystems reach edible crops and compromise their safety and quality. Mechanistically, soil antibiotic residues can reshape rhizosphere microbial communities and disrupt nutrient cycling processes (Keppler et al., 2021; Santás-Miguel et al., 2021; Zhang, Li, et al., 2024), and can be absorbed by edible crops, thereby interfering with its metabolic pathways (Xiang et al., 2022). Consistent with these mechanisms, previous studies have reported that soil antibiotics can alter mineral nutrient accumulation in edible crops (Lung et al., 2025). However, most available evidence has relied on a narrow range of nutritional indicators or biomass-related endpoints, which may not adequately capture overall food quality changes. By comparison, the impacts of soil antibiotic residues on the bioactive composition of edible crops remain far less explored, with limited direct evidence available.

Soybean sprouts are widely consumed in Asia for their high nutritional value and rich bioactive composition (Liu et al., 2023; Qiao et al., 2024). Their short growth cycle, active water uptake, and common consumption in fresh form make them a sensitive and representative model for assessing contaminant uptake and nutritional impacts in soil-grown vegetables (Cao et al., 2022). Key nutrients in soybean sprouts—such as amino acids and vitamin C—accumulate rapidly during germination and are closely linked to both metabolic regulation and dietary quality (Ji et al., 2022; Liu et al., 2021). These nutritional components, together with stress-responsive bioactive composition such as phenolics and flavonoids, are known to be highly sensitive to environmental perturbations. Tetracyclines are among the most widely used veterinary antibiotics and frequently accumulate in agricultural soils at relatively high levels, largely due to the application of animal manure, sewage sludge, and wastewater irrigation (Cela-Dablanca et al., 2022; Wang et al., 2025). While studies have documented tetracycline uptake by crops (Azanu et al., 2020; Cela-Dablanca et al., 2022; Yu et al., 2022), limited evidence suggests that tetracycline exposure may induce secondary metabolic responses in edible crops (Lung et al., 2025), and the potential role of rhizosphere microbiota in mediating antibiotic-induced changes in nutritional quality and bioactive metabolism remains poorly characterized. This knowledge gap limits our understanding of how antibiotic contamination may compromise the quality and health value of edible crops.

Therefore, this study focuses on tetracycline and uses soybean sprouts as a representative soil–vegetable model. By integrating transcriptomic profiling with rhizosphere microbiota analysis, the present work explores the metabolic and microbial processes associated with tetracycline-induced changes in nutritional quality and bioactive composition. Furthermore, correlation analysis between rhizosphere microbial communities and quality-related parameters was conducted to investigate potential microbiota–nutrient and microbiota–bioactive associations. These findings provide new insights into how antibiotic residues compromise the quality of edible crops and may inform strategies for mitigating contamination risks in soil-based production systems.

## 2 Material and methods

### 2.1 Materials and chemicals

Soybeans were obtained from Henan Shangke Seed Industry Co., Ltd. (Henan, China). The soil used for soybean sprout cultivation was collected from a vegetable field in Pidu District, Chengdu, Sichuan Province, China (30.882°N, 103.905°E). Prior to use, the collected soil was screened for background tetracycline residues using the established HPLC method, and no tetracycline antibiotics were detected above the method detection limit. The basic physicochemical properties of the soil were as follows: pH 5.8, organic matter 19.2 g/kg, total nitrogen 11.3 g/kg, total phosphorus 8.5 g/kg, and available nitrogen, available phosphorus, and available potassium of 164.3, 96.1, and 132.6 mg/kg, respectively. Tetracycline (98.0 % purity), bovine serum albumin, and Coomassie Brilliant Blue G-250 were purchased from Solabio Technology Co., Ltd. (Beijing, China). Coumestrol standard (≥99 % purity) was purchased from Merck KGaA (Darmstadt, Germany). Methanol, acetonitrile, and formic acid were obtained from Thermo Fisher Scientific (Shanghai, China). All other chemicals used in this study were of analytical grade.

### 2.2 Cultivation of soybean sprouts and sample treatment

Soybean sprouts were cultivated using the method described by Płonka et al. (2022). Mature, undamaged soybeans were soaked in water at 30 °C for 3 h. The experimental groups were treated with tetracycline at concentrations of 25 mg/kg (TC25) and 50 mg/kg (TC50) to simulate soil antibiotic contamination (based on literature reports of Xu, 2019), while the control group (CON) remained untreated. After soaking, the seeds were sown in seedling trays filled with soil and incubated at 25 °C in darkness for 5 days. Samples were collected daily. The radicles, hypocotyls, and cotyledons were stored at −80 °C for tetracycline residue analysis, while the remaining parts were used for the growth performance and nutritional quality assays. For the measurement of nutritional indicators, multiple soybean sprouts were pooled to generate each composite sample. Rhizosphere soil was collected on the 3rd and 5th days for high-throughput sequencing. Hypocotyls were harvested on the 5th day for subsequent transcriptomic analysis. Each cultivation and sampling process was performed in triplicate.

### 2.3 Tetracycline residue in soybean sprouts

The various organs of soybean sprouts were pretreated following the method described by Gu et al. (2021). Briefly, a certain amount of each sample was placed in a centrifuge tube (Shanghai Titan Scientific Co. Ltd. Shanghai, China) with 20 mL of extraction solution. The extraction solution was prepared by dissolving 12.9 g citric acid monohydrate, 27.5 g disodium hydrogen phosphate dodecahydrate, and 37.2 g disodium EDTA in 1 L of ultrapure water (pH 4.0) to obtain a Na₂EDTA–McIlvaine solution, which was then mixed with methanol at a 1:1 (*v*/v) ratio. The tube was vortexed for 1 min, followed by ultrasonication in a water bath at room temperature for 15 min. Subsequently, the sample was centrifuged at 2300 ×*g* for 15 min (Thermo Fisher Scientific, Waltham, MA, USA), and the supernatant was collected into centrifuge tube. This extraction procedure was repeated three times. The combined supernatants were concentrated to half of the original volume using a rotary evaporator to remove methanol. After a further 15 min of ultrasonication, the final extract was filtered through a 0.22 μm membrane filter to remove particulates, and the filtrate was transferred to amber vials (ANPEL Laboratory Technologies Inc., Shanghai, China). The extract was stored at −20 °C until analysis.

The concentration of tetracycline in the extract was analyzed using a high-performance liquid chromatography (HPLC) system (Waters 2695, Waters Corp., Milford, MA, USA) equipped with an Agilent Eclipse Plus C18 column (4.6 × 150 mm, 5 μm; Agilent Technologies, Santa Clara, CA, USA). The column was maintained at 30 °C, and the flow rate was set at 1.0 mL/min. The injection volume was 20 μL. The mobile phase consisted of solvent A (ultrapure water with 0.1 % formic acid) and solvent B (acetonitrile), with an isocratic elution of 90 % A and 10 % B from 0 to 30 min. UV detection was carried out at 340 nm. Method validation showed that the method detection limit (LOD) and limit of quantification (LOQ) for tetracycline were 0.01 and 0.04 mg/L, respectively, and the spiked recovery ranged from 85.2 % to 111.6 %, with RSD values between 0.7 % and 4.2 %, indicating good analytical accuracy and precision.

### 2.4 Growth performance of soybean sprouts

A total of 120 soybean sprouts were randomly selected from each seedling tray by numbering all sprouts and selecting individuals using a computer-generated random number sequence. The lengths of the hypocotyls and radicles were measured using a calibrated ruler (minimum scale: 0.02 mm; model DL3943, Ningbo Deli Tools Co., Ltd., Ningbo, China). The measurements were expressed as the mean ± standard deviation. For biomass determination, the radicles were removed, and the remaining tissues were oven-dried at 105 °C to a constant weight.

### 2.5 Determination of nutritional quality of soybean sprouts

#### 2.5.1 Cellulose and total soluble sugar

Cellulose content was determined by the anthrone-sulfuric acid method, followed by Que et al. (2018). 2.0 mL of the cellulose pretreated extract was mixed with 0.5 mL of 2 % anthrone reagent in a test tube, followed by the addition of 5.0 mL concentrated sulfuric acid. The mixture was mixed thoroughly and allowed to stand for 12 min at room temperature. Absorbance was measured at 620 nm and a standard curve was constructed using cellulose solutions of known concentrations.

Total soluble sugar content was measured with a method modified from Dien et al. (2019). Samples were extracted with boiling water, the extracts were reacted with 2 % (*w*/*v*) anthrone reagent in concentrated H₂SO₄ and incubated in a water bath for 12 min, cooled to room temperature, and absorbance was measured at 630 nm. A standard curve was established using sucrose and the soluble sugar content was expressed in milligrams per g DW.

#### 2.5.2 Protein and fat

Protein content in soybean sprouts was determined using the Kjeldahl nitrogen determination method, following the procedure described by Qi et al. (2022). Approximately 1.0 g of dried, ground sprout tissue was digested with sulfuric acid in the presence of catalytic agents, followed by neutralization, distillation, and titration using an automatic Kjeldahl analyzer (K1100, Hanon Instruments, Jinan, China). Total nitrogen was quantified and converted to crude protein using a nitrogen-to-protein conversion factor of 6.25. Protein content was expressed as mg/g dry weight.

Fat content was determined using the Soxhlet extraction method in accordance with GB 5009.6–2016. Briefly, the extraction cup was dried and weighed (M₀), and a known mass of dried, ground sample (M₁) was placed into a cellulose thimble and extracted with petroleum ether under continuous reflux. After extraction, the solvent was removed and the cup was dried at 105 °C to constant weight (M₂). Fat content was calculated as follows:

$$\mathrm{Fat}\;\left(\mathrm{mg}/100\;g\;\mathrm{DW}\right)=\frac{M_{2}-M_{1}}{M_{1}}\times 100000$$

#### 2.5.3 Vitamin C

Vitamin C was extracted from 2.0 g of fresh soybean sprouts by homogenizing the tissue with 5 mL of an oxalic acid–EDTA solution (50 mM oxalic acid and 0.2 mM EDTA), and the homogenate was then diluted to 20 mL with the same solution. The mixture was kept in the dark at room temperature for 30 min, then centrifuged at 2300 ×*g* for 10 min at 4 °C. The supernatant was analyzed for Vitamin C content by measuring absorbance at 760 nm, according to the method described by Le et al. (2021). A standard curve was constructed using ascorbic acid (≥99 % purity, Sigma-Aldrich, St. Louis, MO, USA) as the reference standard, and the calibration curve showed a good linear relationship (R^2^ = 0.998). Vitamin C concentration was expressed in milligrams per 100 g dry weight (DW).

#### 2.5.4 Amino acid

The free amino acid content in soybean sprouts was determined using an ultra-performance liquid chromatography–tandem mass spectrometry (UPLC-MS/MS) system equipped with a Cortex UPLC C18 column (50 × 2.1 mm, 1.6 μm; Waters Corporation, Milford, MA, USA) operated at 40 °C with a flow rate of 0.5 mL/min (Zhao et al., 2021). The mobile phase consisted of solvent A (ultrapure water containing 0.1 % formic acid) and solvent B (acetonitrile). The gradient elution program was set as follows: 0.0–0.5 min, 1 % B (isocratic); 0.5–2.0 min, 1–10 % B; 2.0–2.5 min, 10–15 % B; 2.5–3.0 min, 15 % B; 3.0–4.0 min, 15–20 % B; 4.0–4.5 min, 20–99 % B; 4.5–5.0 min, 99 % B; 5.0–5.2 min, 99–1 % B; and 5.2–7.0 min, re-equilibration at 1 % B. The injection volume was 5 μL. Mass spectrometric detection was performed in positive electrospray ionization mode (ESI+) using multiple reaction monitoring. The optimized MS parameters were as follows: gas temperature, 150 °C; sheath gas heater temperature, 550 °C; sheath gas flow rate, 1000 L/h; and capillary voltage, 2000 V.

### 2.6 Determination of bioactive composition of soybean sprouts

#### 2.6.1 Total flavonoid and total phenol

Soybean sprout samples were oven-dried to a constant weight, ground, and passed through a 60-mesh screen. An accurately weighed 0.02 g sample was extracted with 2 mL of 60 % ethanol solution. The mixture was shaken at 60 °C for 2 h, then centrifuged at 11,200 ×*g* for 10 min at room temperature. The supernatant was collected and analyzed using a plant flavonoid and total phenol assay kit (Nanjing Jiancheng Bioengineering Institute, Nanjing, China). following the manufacturer's instructions, respectively. For the total flavonoid assay, rutin was used as the reference standard, and the standard curve exhibited strong linearity (R^2^ = 0.999). For the total phenol assay, gallic acid served as the reference standard, with a calibration curve linearity of R^2^ = 0.999. Flavonoid and total phenol contents were expressed as mg/g dry weight (DW).

#### 2.6.2 Coumestrol

The coumestrol content in soybean sprouts during growth was determined using HPLC (Waters 2695, Waters Corp., Milford, MA, USA) following the method described by Ohta et al. (2021). Chromatographic separation was performed on an Agilent Eclipse Plus C18 column (4.6 × 150 mm, 5 μm; Agilent Technologies, Santa Clara, CA, USA), using a mobile phase consisting of 0.1 % formic acid in water (solvent A) and 100 % acetonitrile (solvent B). Isocratic elution was carried out with 60 % A and 40 % B for 20 min at a flow rate of 1.0 mL/min. The injection volume was 10 μL, and UV detection was set at 343 nm. The column temperature was maintained at 40 °C. A standard curve was constructed using coumestrol standard solutions of known concentrations, and peak areas were plotted against concentration to generate a regression equation. Coumestrol concentrations in the samples were calculated based on this equation and expressed as μg/g dry weight (DW). Method validation showed that the LOD and LOQ for coumestrol were 0.02 and 0.08 mg/L, respectively. The spiked recovery ranged from 88.3 % to 116.2 %, with RSD values between 2.1 % and 5.2 %, indicating good analytical accuracy and precision.

### 2.7 16S rRNA high-throughput sequencing

Total genomic DNA from rhizosphere soil was extracted using a Soil DNA Extraction Kit (Tiangen Biotech Co., Ltd., Beijing, China) according to the manufacturer's instructions. DNA quality and concentration were measured using a NanoDrop 2000 spectrophotometer (Thermo Fisher Scientific, Waltham, MA, USA). The bacteria 16S rRNA gene was amplified using primers 515F (GTGYCAGCMGCCGCGGTAA) and 806R (GGACTACHVGGGTWTCTAAT). PCR was performed in a 50 μL reaction containing 5 μL 10 × PCR buffer, 5 μL dNTPs (0.2 mmol/L), 1 μL KOD-Plus-Neo polymerase, 3 μL MgSO_4_ (1.5 μmol/L), 1.5 μL of each primer (0.3 μmol/L), and 2 μL template DNA. Cycling conditions were: PCR conditions were as follows: 94 °C for 1 min; 30 cycles of 94 °C for 20 s, 54 °C for 30 s, 72 °C for 30 s; and final extension at 72 °C for 5 min. Sequencing was conducted on the Illumina MiSeq PE250 platform. Raw reads were quality-filtered using QIIME (v1.9), merged with FLASH, and clustered into operational taxonomic units (OTUs) at 97 % similarity using UPARSE. Although OTU-based pipelines are less commonly used than QIIME2/DADA2, the dataset was further evaluated using standard ecological metrics. Alpha diversity and beta diversity were calculated, and statistical significance of community differences was tested using PERMANOVA.

### 2.8 RNA sequencing analysis of soybean sprouts

Total RNA was extracted from soybean sprouts after a 5-day treatment with 50 mg/kg tetracycline at 25 °C using the TRIzol method (Invitrogen, Carlsbad, CA, USA). The untreated group served as the control. RNA integrity and concentration were assessed using 1 % agarose gel electrophoresis, a NanoDrop 2000 spectrophotometer (Thermo Fisher Scientific, Waltham, MA, USA), and an Agilent Bioanalyzer 2100 system (Agilent Technologies, Santa Clara, CA, USA). mRNA was enriched from total RNA using Poly-T oligo-attached magnetic beads (Thermo Fisher Scientific, Waltham, MA, USA). The purified mRNA was then fragmented and used to construct a cDNA library, which was sequenced on an Illumina NovaSeq 6000 platform (Illumina Inc., San Diego, CA, USA).

Raw reads were mapped to the soybean sprout genome using Bowtie2 with default parameters. Sequencing quality control metrics, including read depth and base quality values, are provided in Supplementary Table S1. Differential expression between the treatment and control groups was calculated using DESeq2. Genes with a log2(fold change) > 1 were considered as differentially expressed genes (DEGs). Enrichment analysis and functional annotation of the DEGs were then performed using Gene Ontology (GO) resources (http://geneontology.org/) and the Kyoto Encyclopedia of Genes and Genomes (KEGG) pathways (https://www.genome.jp/kegg/).

### 2.9 Statistical analysis

All experiments were performed using three separate biological samples. Each biological sample was analyzed once. Data are presented as mean ± standard deviation (SD) calculated from the biological samples. Statistical analysis was performed using one-way analysis of variance (ANOVA) followed by Tukey's post hoc test in SPSS 23.0 (IBM Corp., Armonk, NY, USA). Before conducting ANOVA, the assumptions of normality and homogeneity of variances were verified using the Shapiro–Wilk test and Levene's test, respectively. Graphs were generated using GraphPad Prism 9.0 (GraphPad Software, San Diego, CA, USA) and R 4.5.1 software (R Foundation for Statistical Computing, Vienna, Austria). The relative abundances of microbial species were correlated with the nutritional parameters of soybean sprouts, *P*-values were adjusted for multiple testing using the Benjamini–Hochberg false discovery rate (FDR) correction. Spearman's rank correlation coefficients were calculated using SPSS 23.0, and the correlation network was visualized using Cytoscape (Cytoscape Consortium, San Diego, CA, USA).

## 3 Result and discussion

### 3.1 Tetracycline residues in soybean sprouts

**Fig. 1:**
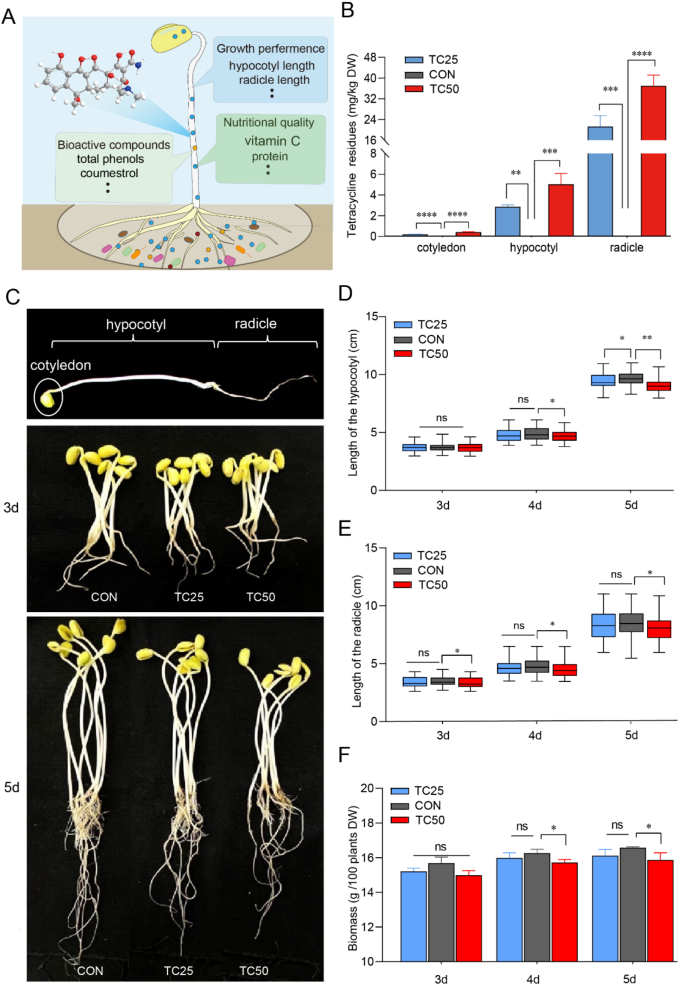
Effect of tetracycline residue on growth performance of soybean sprouts. (A) Overview of tetracycline residue effects in a soil-vegetable system**.** (B) Distribution of tetracycline residue in different soybean sprouts organs at day 5. (C) Growth performance of soybean sprouts. (D) Hypocotyl length. (E) Radicle length. (F) Biomass. CON: control group. ns: not significant (*P* > 0.05), *: *P* < 0.05, **: *P* < 0.01, ***: *P* < 0.001, ****: *P* < 0.0001.

Antibiotics, once they enter agricultural environments such as soil and irrigation water can accumulate in edible crops through absorption, posing potential risks to food safety and human health (Inyinbor et al., 2021; Yu et al., 2022). Among the various antibiotics detected in vegetables such as radish, rapeseed, celery, and coriander, tetracyclines have been found to exhibit the highest concentrations (Hu, Zhou, & Luo, 2010). In the case of soybean sprouts, tetracycline residue from the soil-vegetable system was absorbed and translocated into the radicles, hypocotyls, and cotyledons (Fig. 1A). Our results further showed that the residual tetracycline content in the radicle, hypocotyl, and cotyledon of soybean sprouts increased in proportion to the tetracycline concentration in the soil-vegetable system. Notably, the tetracycline concentration was significantly higher in the radicles than in the hypocotyls and cotyledons (Fig. 1B and Supplementary Table S1). This pattern is consistent with the findings of Pan and Chu (2017), suggesting that the radicles play a critical role in the initial uptake and accumulation of antibiotics from soil-vegetable system. Through the process of transpiration, these compounds are subsequently translocated to other tissues, including the hypocotyls and cotyledons (Goldstein et al., 2014). These results emphasized the importance of understanding the movement of antibiotics within edible crops, as their accumulation in edible parts could pose potential health risks to humans.

### 3.2 Effect of tetracycline residue on the growth performance of soybean sprouts

Research has demonstrated that antibiotic residues remaining in vegetables may potentially affect their growth and development (Sun et al., 2021). Tetracycline residue significantly inhibited the growth performance of soybean sprouts, with the inhibitory effect becoming more pronounced as the tetracycline concentration increased (Fig. 1C). On day 5 of growth, the TC25 (*P* < 0.05) and TC50 groups (*P* < 0.01) showed a significant reduction in hypocotyl length compared to CON group (Fig. 1D), with the TC50 group also exhibiting a marked decrease in radicle length on both day 3–5 (*P* < 0.05) (Fig. 1E). Additionally, high concentrations of tetracycline residue led to a reduction in the biomass of soybean sprouts on both day 4 and day 5 (*P* < 0.05) (Fig. 1F). Although no universal tissue-level toxic threshold for tetracycline has been established, studies in oilseed rape have shown minimal phytotoxicity at concentrations ≤1 mg/kg but clear growth inhibition when exposure exceeds 50 mg/kg (Kacienė et al., 2024). Likewise, tissue accumulations of several to tens of μg/g have been associated with growth suppression in vegetables (Liu et al., 2018).

Previous studies have shown that antibiotic residues in soil generally exert a “low-promotion, high-inhibition” effect on edible crop growth, with specific responses varying depending on antibiotic type and crop species (Han et al., 2019). A synthesis of soil antibiotic studies indicates that experimentally simulated residue concentrations commonly range from 20 to 100 mg/kg, within which biological effects on soil–plant systems are frequently observed (Grenni et al., 2018). Focusing on tetracycline, Xu (2019) investigated a gradient of soil concentrations (1–100 mg/kg) and demonstrated that edible crop growth was not significantly affected below 25 mg/kg, began to respond at approximately 25 mg/kg, and was markedly inhibited at 50 mg/kg or higher. These results suggest that tetracycline concentrations around 25 mg/kg represent a biological response threshold, whereas concentrations ≥50 mg/kg fall within a clear inhibitory range. Accordingly, the concentrations selected in this study (25 and 50 mg/kg) were designed to represent environmentally relevant threshold and inhibitory levels of soil tetracycline residues. Mechanistically, low concentrations of antibiotics may stimulate soil microbial activity and organic carbon mineralization, whereas higher concentrations suppress microbial processes and edible crop growth (Zhang, Han, et al., 2024). In addition, species-specific differences in antibiotic uptake and metabolism can further modulate growth responses, contributing to the variable sensitivity observed among different edible crops (Azanu et al., 2020; Sun et al., 2021).

### 3.3 Effect of tetracycline residue on nutritional quality of soybean sprouts

#### 3.3.1 Basic nutritional composition of soybean sprouts

Soybean sprouts are widely consumed due to their high nutritional value and short growth period (Qiao et al., 2024). We examined the effects of tetracycline residue in the soil-vegetable system on basic nutritional components of soybean sprouts, including cellulose, protein, total soluble sugars, and fat. Tetracycline showed minimal influence on protein and fat contents, and only a transient increase in total soluble sugars was observed on day 3 in TC25 and TC50 (Table 1). In contrast, cellulose content responded sensitively to tetracycline exposure. Cellulose levels were consistently higher across the germination period, with a more pronounced increase at lower tetracycline concentrations (*P* < 0.05). On day 5, cellulose content in TC25 (374.9 ± 18.3 mg/100 g DW) and TC50 (336.4 ± 11.3 mg/100 g DW) was elevated by 22.4 ± 1.7 % and 9.9 ± 0.7 %, respectively, compared with the control group (306.2 ± 18.2 mg/100 g DW). As cellulose is a major structural and nutritional component of soybean sprouts, its accumulation is often influenced by nutrient uptake and carbon allocation patterns. Previous studies have shown that antibiotic residues can alter soil microbial diversity and the activities of key soil enzymes, such as phosphatases, nitrate reductase and cellulase (Cycoń et al., 2019; Santás-Miguel et al., 2021; Wang et al., 2024). These microbial and enzymatic changes are known to affect the availability of essential nutrients (e.g., nitrate, potassium), which are closely linked to cell-wall biosynthesis in edible crops. Thus, the sustained increase in cellulose observed here is consistent with nutrient-availability shifts reported under antibiotic stress, and may reflect an adjustment in carbon allocation toward structural carbohydrates in soybean sprouts (Nkoh et al., 2024).

**Table 1:** Effects of tetracycline residues on the basic nutritional composition of soybean sprouts.

Parameters	Groups	The growth period of soybean sprouts (days)		
		3	4	5
Cellulose (mg/g dry weight)	CON	158.6 ± 15.4^a^	251.6 ± 14.5^a^	306.2 ± 18.2^a^
	TC25	187.8 ± 9.9^b^	219.3 ± 16.2^b^	374.9 ± 18.3^b^
	TC50	200.4 ± 12.3^b^	188.2 ± 12.5^c^	336.4 ± 11.3^b^
Protein (mg/g dry weight)	CON	376.6 ± 13.3^a^	390.2 ± 14.2^a^	386.3 ± 9.2^a^
	TC25	366.6 ± 13.3^a^	400.2 ± 27.4^a^	392.2 ± 10.5^a^
	TC50	385.1 ± 10.2^a^	389.5 ± 12.1^a^	389.6 ± 13.1^a^
Total Soluble sugar (mg/g dry weight)	CON	129.6 ± 6.4^a^	138.3 ± 11.3^a^	187.2 ± 8.0^a^
	TC25	142.6 ± 5.7^b^	128.1 ± 9.3^a^	183.7 ± 6.9^a^
	TC50	156.2 ± 5.6^b^	125.0 ± 10.2^a^	176.9 ± 9.6^a^
Fat (mg/100 g dry weight)	CON	18.5 ± 0.4^a^	17.2 ± 0.2^a^	16.8 ± 0.2^a^
	TC25	18.1 ± 0.3^a^	17.1 ± 0.2^a^	16.5 ± 0.3^a^
	TC50	17.7 ± 0.2^a^	16.7 ± 0.5^a^	16.3 ± 0.3^a^

Note: Different letters indicate significant differences from the CON group (*P* < 0.05).

#### 3.3.2 Amino acid composition

**Fig. 2:**
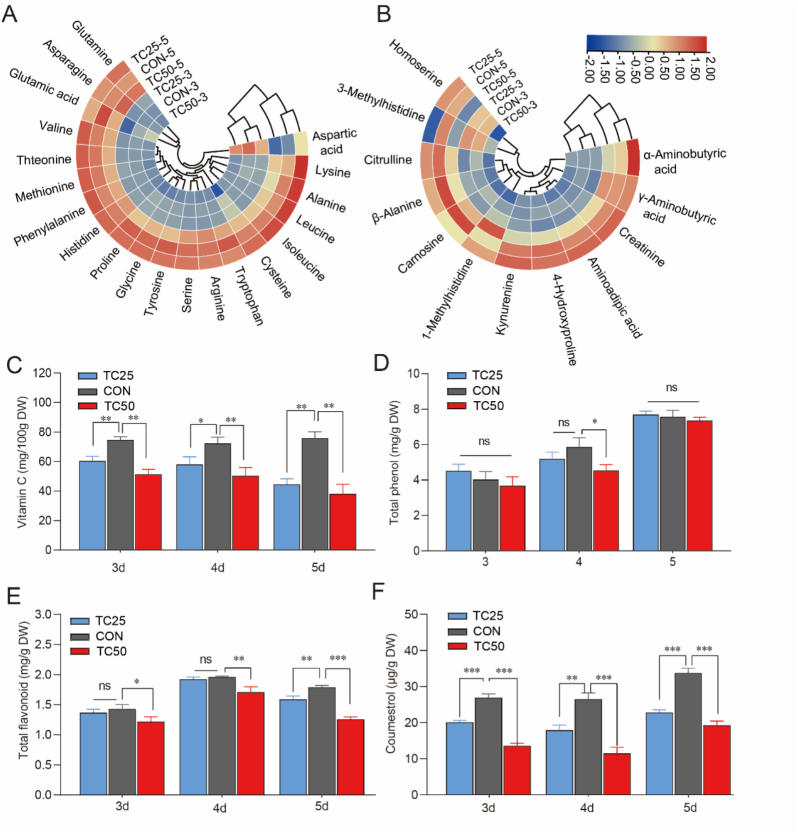
Effects of tetracycline residue on amino acid profiles, vitamin C, and bioactive composition of soybean sprouts. (A) Heatmaps of amino acids. (B) Heatmaps of amino acid derivatives. (C) Vitamin C. (D) Total phenol. (E) Total flavonoid. (F) Coumestrol. ns: *P* > 0.05, *: *P* < 0.05, **: *P* < 0.01, ***: *P* < 0.001.

Soybean sprouts contain a variety of essential amino acids, which play an important role in determining the nutritional quality. During the growth of soybean sprouts, stored proteins are hydrolyzed, subsequently releasing bioactive amino acids and peptides, leading to continuous changes in the amino acid content (Bueno et al., 2020). Overall, the total amino acid content continuously increased during the growth of soybean sprouts. In TC25 group, the content of proline, tryptophan and glutamic acid decreased, while the levels of leucine, isoleucine, alanine, and lysine increased at day of 5. In contrast, exposure to 50 mg/kg tetracycline resulted in a significant reduction in most amino acids, with notable decreases in glutamic acid, proline, tryptophan and so on (Fig. 2A). Similarly, tetracycline residue also significantly affected the content changes of amino acid derivatives. And exposure to 25 mg/kg tetracycline caused a rise in 1-methylhistidine and α-aminobutyric acid, while 3-methylhistidine, β-alanine, and carnosine levels decreased. At 50 mg/kg tetracycline, 1-methylhistidine levels increased, while the levels of other amino acid derivatives decreased, including 3-methylhistidine, β-alanine, 4-hydroxyproline, α-aminoadipic acid, and creatinine (Fig. 2B).

These findings revealed that tetracycline residues not only disrupt the intrinsic antioxidant system of soybean sprouts but also affect their nitrogen metabolism cycle. Carnosine, synthesized from β-alanine as a precursor, is an important bioactive compound with various biological functions, including antioxidant, anti-aging, and anti-inflammatory properties (Wu et al., 2003), which significantly enhanced the antioxidant capacity and nutritional value of soybean sprouts. Additionally, previous study reported 4-hydroxyproline and 3-methylhistidine contributed to reducing the accumulation of free radicals (Wu, 2020), further enhancing the antioxidant capacity of soybean sprouts. However, tetracycline residue significantly inhibits the synthesis of these compounds (Fig. 2B), leading to a decrease in the antioxidant capacity of soybean sprouts. The et al. (2021). reported that environmental stress can disrupt the nitrogen metabolism cycle, which may explain the significant reduction of nitrogen-related metabolites such as α-aminobutyric acid, aminoadipic acid, and creatinine observed in soybean sprouts.

#### 3.3.3 Vitamin C contents

Vitamin C plays a critical role in human health as a potent antioxidant, immune system booster, and a key nutrient in preventing scurvy (Ji et al., 2022; Liu et al., 2021). Our results showed that tetracycline residue exhibited a significant inhibitory effect on the synthesis of vitamin C in soybean sprouts (*P* < 0.01), with the degree of inhibition becoming more pronounced as the tetracycline concentration increased. On day 5, the vitamin C content in the TC25 (44.5 ± 3.8 mg/100 g DW) and TC50 groups (38.1 ± 6.6 mg/100 g DW) was reduced by 41.3 ± 2.6 % and 49.8 ± 4.3 %, respectively, compared to the CON group (75.9 ± 4.3 mg/100 g DW) (*P* < 0.01) (Fig. 2C). This decline may result from increased oxidative stress induced by antibiotic exposure (Li et al., 2024), which accelerates vitamin C depletion and disrupts its redox homeostasis. These findings highlight potential risks to the nutritional quality of sprouts cultivated in antibiotic-contaminated environments.

### 3.4 Effect of tetracycline residue on bioactive composition of soybean sprouts

Soybean sprouts contain abundant bioactive composition such as flavonoids, phenolics, and coumestrol (Zhang et al., 2019). Tetracycline residue did not alter total phenolic content (Fig. 2D), but it significantly suppressed total flavonoids and coumestrol (Fig. 2E–F). In the TC25 group, total flavonoid levels remained unchanged during early germination but declined significantly on day 5, decreasing by 11.1 ± 3.4 % compared with the control (*P* < 0.01). In contrast, TC50 caused a consistent inhibition throughout the growth period, resulting in a 29.7 ± 2.5 % reduction on day 5. Isoflavones—including genistein, daidzein, and glycitein—are the major flavonoids in soybean sprouts and contribute to their antioxidant and anti-inflammatory properties (Kim & Kim, 2020; Qiao et al., 2024). Given that flavonoids and coumestrol share closely related biosynthetic pathways, alterations in flavonoid metabolism may also influence coumestrol accumulation. Coumestrol, which shares the precursor p-coumaroyl-CoA with flavonoids (Oshima et al., 2016), was even more sensitive to tetracycline exposure. Both TC25 (22.8 ± 0.8 μg/g DW) and TC50 (19.3 ± 1.1 μg/g DW) caused significant suppression across all growth stages, with coumestrol levels on day 5 decreasing by 32.43 ± 1.6 % and 42.9 ± 3.0 %, respectively, compared with the control (33.7 ± 1.3 μg/g DW) (Fig. 2F). Antibiotic residues in soil can influence the synthesis of bioactive composition in edible crops. They may disrupt normal metabolic processes by interfering with key enzymatic systems (Batuman et al., 2024). In addition, antibiotics can impair hormonal regulation, thereby reduce the activity of secondary metabolic pathways and subsequently limit the synthesis of bioactive composition (Opriş et al., 2013).

### 3.5 Changes in the rhizosphere microbial community of soybean sprouts

**Fig. 3:**
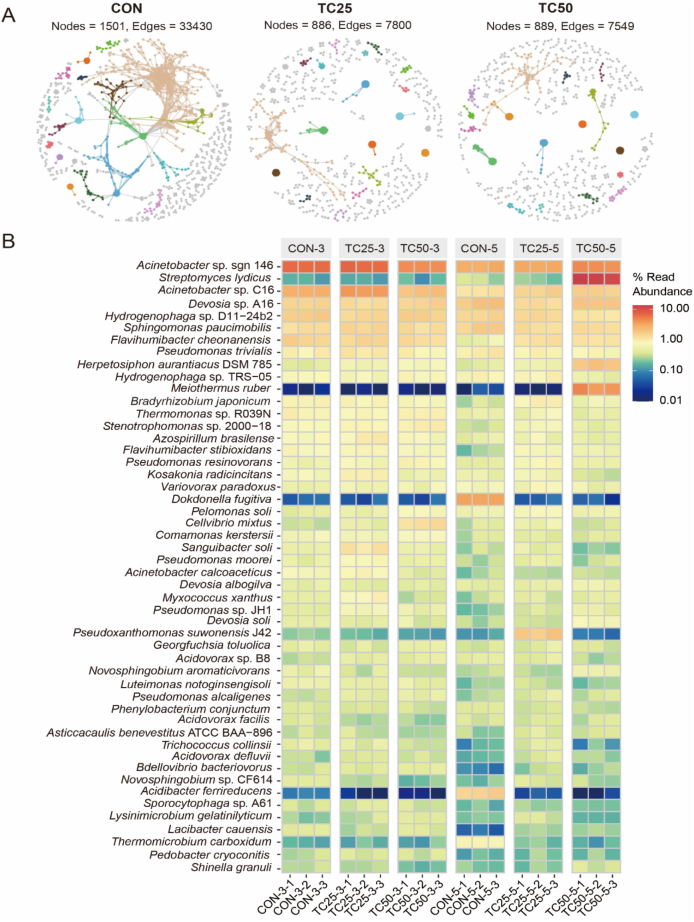
Effect of tetracycline residue in soil on the rhizosphere bacteria of soybean sprouts. A: The cooccurrence networks of bacteria in different groups. The nodes are colored to indicate different bacterial modules, the correlations were inferred from OTUs abundance profiles using the spearman method and only the robust and significant (correlation values <−0.7 or > 0.7 and *P* < 0.05) correlations were maintained for the construction of co-occurrence networks, each node corresponds to the bacterial OTUs, the statistical test used was two-sided. B: Heatmaps based on bacterial species level.

Rhizosphere microbial communities are a crucial component of the soil ecosystem, playing a key role in supporting the growth and maintaining edible crops health (Luo et al., 2025). Antibiotic residues in soil-vegetable system can selectively target specific microbes, thereby altering the diversity, structure, and functionality of the rhizosphere microbial community (Underwoodu et al., 2011). Our results demonstrated that tetracycline residue significantly altered the composition of rhizosphere microbial communities of soybean sprouts (Fig. 3). The cooccurrence networks of bacteria revealed that tetracycline residue significantly weakened the interactions among bacteria in the rhizosphere, leading to a notable decrease in the number of key nodes (Fig. 3A). This disruption was likely to cause an imbalance in the microbial community, which could negatively affect the growth and quality of soybean sprouts. Previous studies have shown that tetracycline residual can inhibit the abundance of soil bacteria and actinobacteria, directly reducing microbial biomass, the inhibitory effect was more pronounced at higher concentrations of antibiotic contamination (Santás-Miguel et al., 2021). On day 3 of soybean sprout growth, tetracycline residue did not significantly impact the dominant bacterial species, however, a significant shift in the dominant bacterial populations was observed by day 5. The dominant bacterial genera in the CON group were *Acinetobacter* sp. sgn 146, *Dokdonella fugitiva* and *Acidibacter ferrireducens*, the dominant genera were *Acinetobacter* sp. sgn 146, *Acinetobacter* sp. C16 and *Pseudoxanthomonas suwonensis* J42 in the TC25 group, the dominant genera were *Streptomyces lydicus*, *Acinetobacter* sp. sgn 146 and *Meiothermus ruber* in the TC50 group (Fig. 3B).

Specifically, the relative abundances of *Acinetobacter* sp., *Meiothermus ruber* and *Streptomyces lydicus* were significantly increased in tetracycline treatment. These taxa are known for their robust environmental adaptability. As potential hosts of antibiotic resistance genes, *Acinetobacter* has developed resistance to antibiotic environments through the efflux pump mechanism (Zhou et al., 2022). *Streptomyces lydicus*, as a natural antibiotic producer, likely benefits from intrinsic resistance mechanisms such as efflux pumps, enabling it to thrive under antibiotics stresses (Sánchez-Hidalgo et al., 2020). In contrast, the relative abundances of *Dokdonella fugitive*, *Pseudoxanthomonas suwonensis*, and *Acidovorax defluvii* were markedly reduced (Fig. 3B). These taxa are typically associated with nutrient cycling, organic matter degradation (Hu et al., 2024). Their decline suggests that tetracycline exposure disrupts sensitive functional guilds, likely through selective pressure or competitive exclusion by resistant taxa. These microbial shifts indicate a transition toward a more antibiotic-resistant but potentially functionally impaired rhizosphere community. Overall, tetracycline residual significantly altered the composition of rhizosphere microbiota of soybean sprouts, particularly affecting microorganisms directly or indirectly involved in nitrogen cycling, such as *Acinetobacter* sp., *Dokdonella fugitive*, *Pseudomonas* spp., *Hydrogenophaga* spp. and so on.

### 3.6 Correlations between rhizosphere microbiota and growth performance, nutritional quality, and bioactive composition

**Fig. 4:**
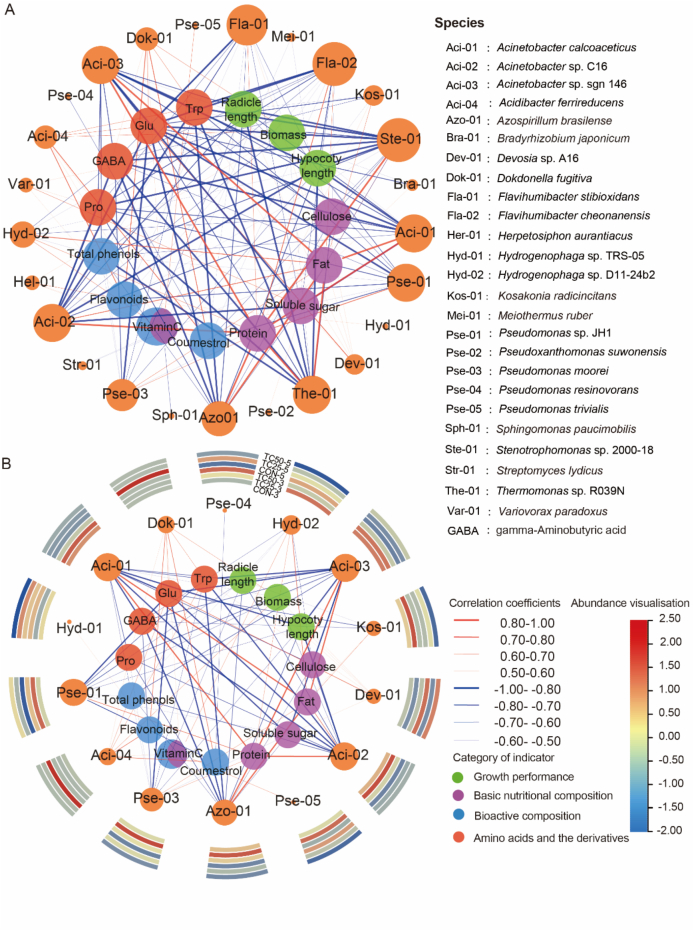
Association between species-level relative abundance of rhizobacterial communities and measured parameters during soybean sprout growth. (A) The correlation network analysis was conducted on 25 bacterial species with high relative abundance and significant inter-group differences, in relation to the measured parameters. (B) A focused subnetwork analysis was further performed on nitrogen-cycling-related species, accompanied by a corresponding heatmap illustrating their relative abundance. The networks were constructed in Cytoscape (v3.10.0) based on Spearman correlation coefficients (calculated using SPSS 23). *P*-values were adjusted for multiple testing using the Benjamini–Hochberg false discovery rate (FDR) correction, and only FDR-adjusted *p* < 0.05 correlations were retained. In these networks, nodes represent variables (bacterial species or measured parameters), color-coded according to category. Edges represent significant correlations (p < 0.05), with red indicating positive correlations (*r* > 0) and blue indicating negative correlations (*r* < 0). The width of each edge is proportional to the strength of the correlation. Relative abundance heatmaps were generated using TBtools. (For interpretation of the references to color in this figure legend, the reader is referred to the web version of this article.)

Rhizosphere microorganisms directly influence the accumulation of nutritional compounds in soybean sprouts by promoting specific physiological and metabolic pathways, such as nutrient absorption, nitrogen fixation, which play a vital role in determining their nutritional quality and bioactive composition (Shi et al., 2025). Considering both significant responses to antibiotic concentrations and relative abundance, 25 microbial taxa were selected for correlation analysis with soybean sprout growth performance, nutritional quality, and bioactive composition. In general, most rhizosphere microbiota showed positive correlations with fat and protein contents in soybean sprouts, while exhibiting negative correlations with secondary metabolites including γ-aminobutyric acid (GABA), flavonoids, total phenols and coumestrol (Fig. 4A). These results indicated that the rhizosphere microbial community was significantly changed under tetracycline stress, the soybean sprouts appeared to prioritize the maintenance of nutrients associated with growth and primary metabolism, which showed positive correlations with fat and protein contents (Fig. 4A). In contrast, the accumulation of functional nutrients associated with stress response and secondary metabolism, such as GABA, flavonoids, vitamin C and coumestrol, was suppressed, exhibiting negative correlations. This reflects a metabolic reallocation in response to environmental stressors, characterized by an enhancement of primary metabolism and a concurrent reduction in secondary metabolism (Salam et al., 2023).

Nitrogen metabolism plays a crucial role in plant growth and essential metabolic processes (Gaudinier et al., 2018). In precent study, we focused on investigating the correlation between nitrogen cycle-related microorganisms and related parameters, it was found that *Dokdonella fugitiva* and *Acidibacter ferrireducens* both showed positive correlations with flavonoids, coumestrol, and vitamin C (Fig. 4B). Microorganisms of the genus *Dokdonella* are mainly responsible for nitrite transformation, thereby promoting edible crops absorption of nitrogen sources (Wang et al., 2019). *Acidibacter ferrireducens* is primarily involved in iron reduction (Falagán & Johnson, 2014), although it does not directly participate in nitrogen metabolism, it indirectly promotes vegetable nitrogen uptake through the action of iron ions (Slimani et al., 2023). Overall, *Dokdonella fugitiva* and *Acidibacter ferrireducens*, as beneficial rhizosphere microbes, are generally associated with improved nitrogen availability and may support the synthesis of secondary metabolites in edible crops. In this study, the marked reduction of these taxa under tetracycline stress was associated with shifts in nitrogen-related metabolic pathways, which in turn corresponded to decreased flavonoid and coumestrol levels. Strains of the *Acinetobacter* genus, including *A. calcoaceticus* and *Acinetobacter* sp., showed negative correlations with radicle length, hypocotyl length, biomass, tryptophan, proline, and γ-aminobutyric acid in soybean sprouts (Fig. 4B). Although certain *Acinetobacter* strains have been reported to enhance vegetable growth (e.g., *Acinetobacter* sp. SW5 promoting tomato seedling growth; Kwon & Song, 2014), their increased abundance under high antibiotic concentrations may play a different ecological role. A plausible interpretation is that, under tetracycline stress, the enrichment of *Acinetobacter* strains co-occurs with intensified competition for nutrients and water among rhizosphere microorganisms (Lazcano et al., 2021), which is associated with reduced soybean sprout growth and lower levels of stress-related metabolites such as tryptophan and proline (Fig. 2A). These associations suggest that tetracycline-induced microbial shifts may influence edible crops metabolic responses, although direct causal relationships remain to be further investigated.

### 3.7 Functional enrichment analysis of the differential genes

**Fig. 5:**
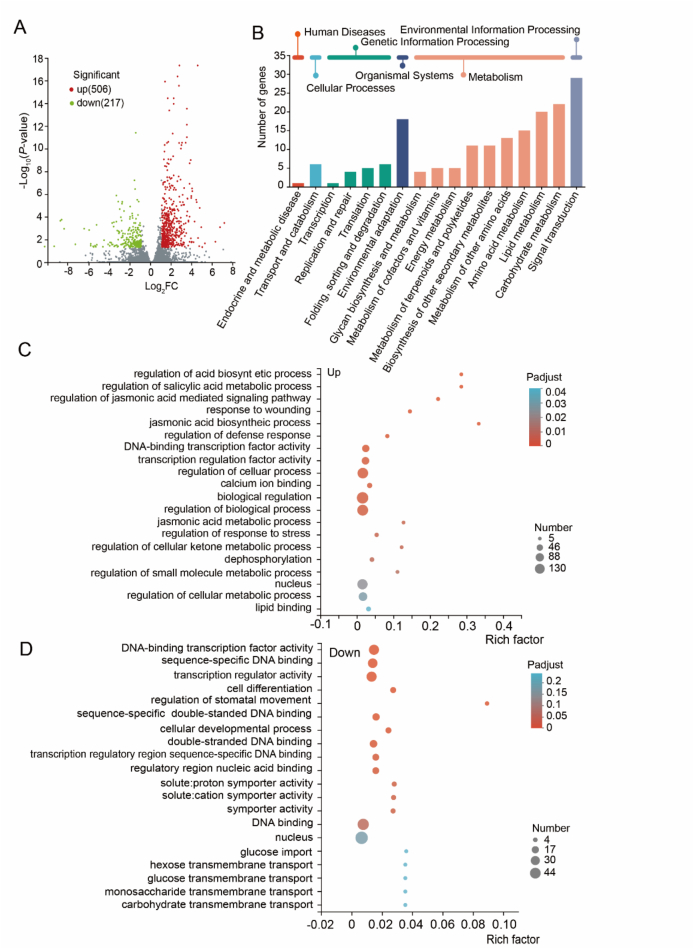
Overview of the differential gene expression analysis (TC50–5 vs CON-5). (A) Volcano plot showing the differentially expressed genes between CON-5 and TC50–5. (B) KEGG pathway annotation results for CON-5 and TC50–5, focuses on broad categories of gene functions. (C) GO enrichment analysis for upregulated differential genes. (D) GO enrichment analysis for downregulated differential genes.

The above results demonstrated that tetracycline residue significantly affected the nutritional quality and bioactive composition of soybean sprouts in a dose-dependent manner (Fig. 1 and Fig. 2; Table 1). Because the 50 mg/kg treatment consistently induced the most pronounced responses across multiple indicators, including hypocotyl length, radicle length, biomass, vitamin C, total phenols, flavonoids, and coumestrol, transcriptomic analysis was therefore performed on sprouts exposed to 50 mg/kg tetracycline to elucidate the underlying molecular mechanisms. A total of 723 differentially expressed genes (DEGs) were identified, including 506 upregulated and 217 downregulated genes (Fig. 5A). KEGG enrichment revealed that many DEGs were associated with signal transduction, carbohydrate and amino acid metabolism, lipid metabolism, and environmental adaptation (Fig. 5B). Upregulated genes were mainly enriched in mitogen-activated protein kinases (MAPK) signaling, plant–pathogen interactions, and taurine and hypotaurine metabolism (Fig. S1), indicating enhanced stress responses. In contrast, downregulated genes were concentrated in isoflavone biosynthesis, consistent with the observed reductions in flavonoids and coumestrol (Fig. 2C).

GO annotation showed that 649 DEGs were classified into biological processes, cellular components, and molecular functions (Fig. S2). GO analysis further showed that upregulated DEGs were involved in jasmines acid related signaling, wound response, and defense processes (Fig. 5C), whereas downregulated DEGs were linked to transcriptional regulation, cell differentiation, stomatal movement, and other developmental functions (Fig. 5D). These patterns indicate a trade-off between stress defense activation and growth-related processes under tetracycline exposure. The induction of jasmines acid and calcium-mediated signaling pathways reflects typical plant responses to environmental stresses (Aftab & Roychoudhury, 2021; Ali & Baek, 2020), suggesting that tetracycline residue is perceived as a xenobiotic stressor.

### 3.8 Mechanisms of tetracycline residue impact on the nutritional quality and bioactive composition of soybean sprouts

**Fig. 6:**
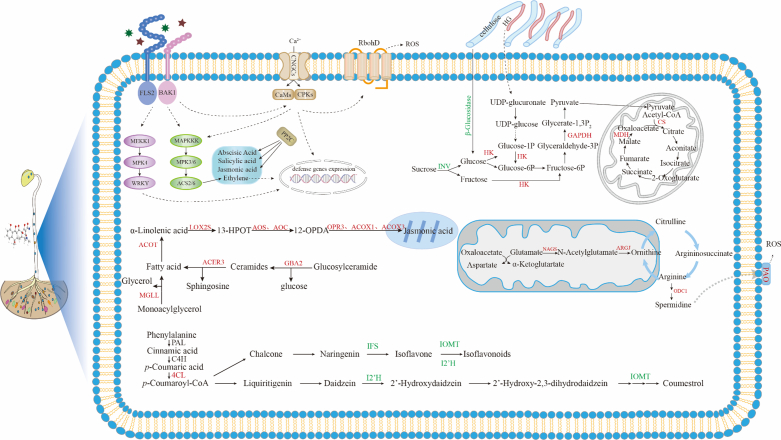
Effect mechanism of tetracycline residue on soybean sprout quality. CNGCs, cyclic nucleotide-gated cation channels. CaMs, calmodulins. PP2C, protein phosphatase. FLS2, Flagellin sensing 2. BAK1, BRI1-associated receptor kinase 1. CPKs, Calcium-dependent protein kinases. RbohD, Respiratory burst oxidase homolog D. ROS, Reactive oxygen species. INV, Invertase. HK, Hexokinase. GAPDH, Glyceraldehyde-3-phosphate dehydrogenase. MDH, Malate dehydrogenase, CS, Citrate synthase. NAGS, N-Acetylglutamate synthase. ARGJ, ‌Acetylornithine deacetylase. ODC1, Ornithine decarboxylase 1. PAO, Polyamine oxidase. LOX2S, Lipoxygenase 2, Short isoform. AOS, Allene oxide synthase. AOC, Allene oxide cyclase. OPR3, *12-*Oxophytodienoate Reductase 3. ACOX1, Acyl-CoA Oxidase1. ACOX3, Acyl-CoA Oxidase 3. GBA2, non-lysosomal glucosylceramidase 2. ACER3, Alkaline ceramidase 3. ACOT, Acyl-CoA thioesterase. MGLL, Monoacylglycerol lipase. 4CL, ‌4-Coumarate-coA ligase‌. IFS, Isoflavone synthase. I2’H, Isoflavone 2′-hydroxylase. IOMT, Isoflavone *o*-methyltransferase.

Antibiotics present in soil can be absorb by edible crops and perturb gene expression and metabolic pathways, ultimately disrupting normal physiological functions. Transcriptomic analysis, a widely used tool for characterizing plant stress responses (Waditee-Sirisattha & Kageyama, 2023), has also revealed stress response mechanisms in other organisms such as *Saccharomyces cerevisiae* under tannic acid exposure (Li et al., 2023). In this study, transcriptomics combined with qPCR validation (Fig. S3) was used to elucidate how tetracycline residue affects the nutritional quality and bioactive composition of soybean sprouts. Tetracycline residue activated multiple stress-response pathways in soybean sprouts (Fig. 6). Upregulation of CNGC and CaM genes suggested enhanced Ca^2+^ influx and calcium signaling, a central regulator of abiotic and biotic stress responses (Liu et al., 2017; Pan et al., 2019). The downregulation of PP2C may have released inhibition on SnRK2 and MAPK signaling, thereby activating ABA-mediated adaptive pathways (Wang et al., 2020). Concurrent induction of FLS2 and MEKK1 indicated activation of PAMP-triggered immunity (Lu et al., 2010; Zhang et al., 2010). GO enrichment further confirmed broad transcriptional activation of stress- and defense-related processes, including jasmonic acid signaling, wounding response, and defense regulation (Fig. 5C). Together, these responses suggest that tetracycline residue is perceived as a xenobiotic stressor, triggering a strong defense program, albeit with potential trade-offs for growth.

Carbon metabolism was also remodeled under tetracycline exposure. Although soluble sugar levels changed little, cellulose content increased (Table 1), consistent with downregulation of *sac*A and β-glucosidase, which may reduce sucrose and glucoside hydrolysis (Singh et al., 2016). In contrast, upregulation of HK, GAPDH, MDH, and CS suggested enhanced glycolysis and TCA cycle flux (Fig. 6), likely supporting energy demands required for stress adaptation (Sun et al., 2018). Amino acid metabolism was substantially altered despite unchanged total protein levels (Fig. 2A, B and Table 1). Tetracycline significantly affected glutamate-associated pathways, which supply precursors for ornithine, arginine, proline, and polyamine biosynthesis, all critical for development and stress responses (Majumdar et al., 2016). Lipid metabolism also participated in the stress response. Although fat content remained stable (Table 1), genes involved in α-linolenic acid metabolism and sphingolipid biosynthesis were upregulated (Fig. 6). Increased expression of LOX2S, AOS, AOC, OPR, and ACOX genes may enhance jasmonic acid production, potentially inhibiting radicle growth (Wasternack & Strnad, 2018). Upregulation of GBA2, ACER3, MGLL, and ACOT suggests increased sphingosine and unsaturated fatty acid production, enhancing membrane fluidity but also potentially inducing growth arrest or apoptosis (Hu, Xu, et al., 2010; Zhao et al., 2018). Secondary metabolism was markedly suppressed. Tetracycline exposure downregulated key genes in the flavonoid and isoflavonoid biosynthesis pathways, consistent with reduced flavonoid and coumestrol levels (Fig. 2E–F). Upregulation of 4CL and CCR in the lignin pathway—known to compete with flavonoid biosynthesis (Dardick et al., 2010)—likely further inhibited flavonoid accumulation (Chen et al., 2021). Downregulation of IFS, I2’H, and IOMT suppressed the synthesis of flavonoid precursors and enzymes essential for coumestrol biosynthesis (Ha et al., 2019; Mochida et al., 2017).

## 4 Conclusion

Overall, tetracycline residue altered the growth and nutritional quality of soybean sprouts. High concentrations of tetracycline significantly reduced the contents of vitamin C, flavonoids, coumestrol, several amino acids, and their derivatives. Transcriptomic analysis revealed disruptions in key metabolic pathways, including carbon metabolism, amino acid metabolism, lipid metabolism, and the biosynthetic pathways of flavonoids and coumestrol. Rhizosphere microbiota analysis showed that tetracycline residue notably affected the abundance of nitrogen-cycling-related microorganisms, with an enrichment of antibiotic-resistant genera (e.g., *Acinetobacter*) and a decline in beneficial taxa (*Dokdonella*, *Acidibacter*). Correlation analysis further demonstrated that these microbial shifts were strongly associated with nutrient loss and growth inhibition. Although the concentrations used here exceed typical environmental levels, they reflect potential contamination scenarios associated with antibiotic misuse, manure over-application, or localized accumulation in agricultural soils. The observed nutritional decline and enrichment of antibiotic-resistant bacteria also align with current regulatory concerns regarding antibiotic residues in crop-production environments. Together, these findings provide integrated transcriptomic and microbial evidence that tetracycline residue compromises the nutritional quality and bioactive composition of soybean sprouts by disrupting microbial composition and important metabolic functions.

## CRediT authorship contribution statement

**Ting Cai:** Writing – review & editing, Project administration, Funding acquisition, Formal analysis. **Jie Yao:** Writing – original draft, Validation, Methodology, Data curation. **Hongmei Jiang:** Writing – original draft, Validation, Investigation, Formal analysis. **Jie Zou:** Methodology, Formal analysis. **Ting Xia:** Data curation. **Xinyue Mou:** Data curation. **Shan Zhang:** Methodology, Data curation. **Xiao Tan:** Writing – review & editing, Supervision. **Jie Tang:** Writing – review & editing, Supervision. **Wenliang Xiang:** Writing – review & editing, Supervision, Project administration, Funding acquisition, Conceptualization.

## Declaration of competing interest

The authors declare that they have no known competing financial interests or personal relationships that could have appeared to influence the work reported in this paper.

## Data Availability

Data will be made available on request.

## Appendix A Supplementary data

Supplementary material Image 1
