# Supplementary material for: Tetracycline residue alters the nutritional quality and bioactive composition of soybean sprouts: Evidence from transcriptomic and rhizosphere microbiota analyses
Source: Food Chem (Oxf). 2025 Dec 31;12:100345. doi: 10.1016/j.fochms.2025.100345 (PMC12810575; doi:10.1016/j.fochms.2025.100345)
Supplement: Supplementary file 1 — Supplementary material [file mmc1.docx]

**Supplementary figure legends**

Fig. S1. KEGG enrichment results of CON-5 group and TC50-5 group, specifically focuses on the relationship between gene expression and KEGG pathways, highlighting upregulated and downregulated genes associated with those pathways.

Fig. S2. GO annotation results of CON-5 group and TC50-5 group, focuses on broad categories of gene functions

Fig. S3. Validation of transcriptome sequencing results by RT-qPCR

Fig. S4. Effects of tetracycline residues on the synthesis of flavonoids and coumestrol of soybean sprouts during growth. 4CL, 4-coumarate-CoA ligase; CCR, cinnamate-CoA reductase; IFS, Isoflavone synthase. I2'H, Isoflavone 2'-hydroxylase. IOMT, Isoflavone o-methyltransferase.

Table S1. Sequencing data quality control for RNA-seq

Table S2. Tetracycline residues in different organs of soybean sprouts


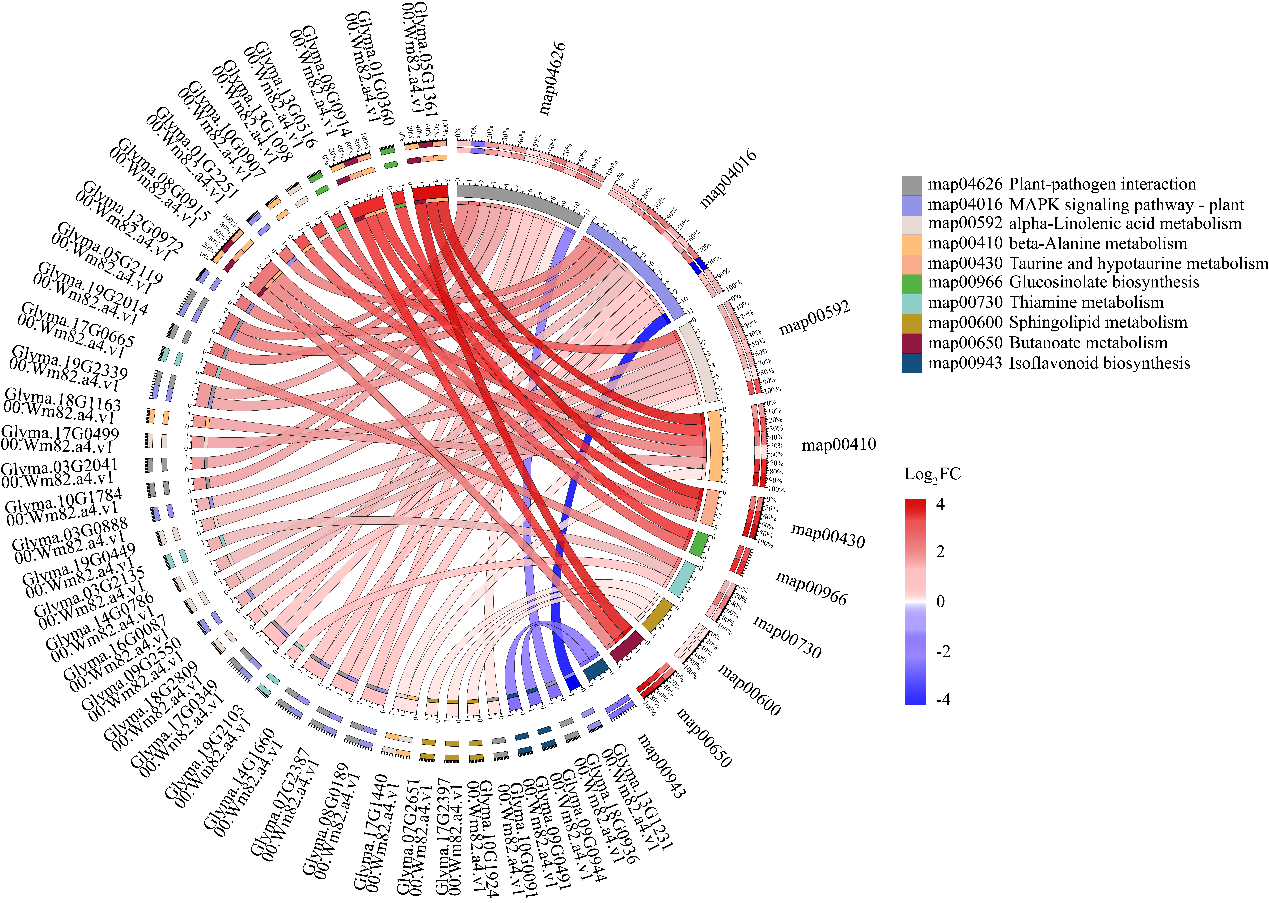
Fig. S1. KEGG enrichment results of CON-5 group and TC50-5 group, specifically focuses on the relationship between gene expression and KEGG pathways, highlighting upregulated and downregulated genes associated with those pathways.


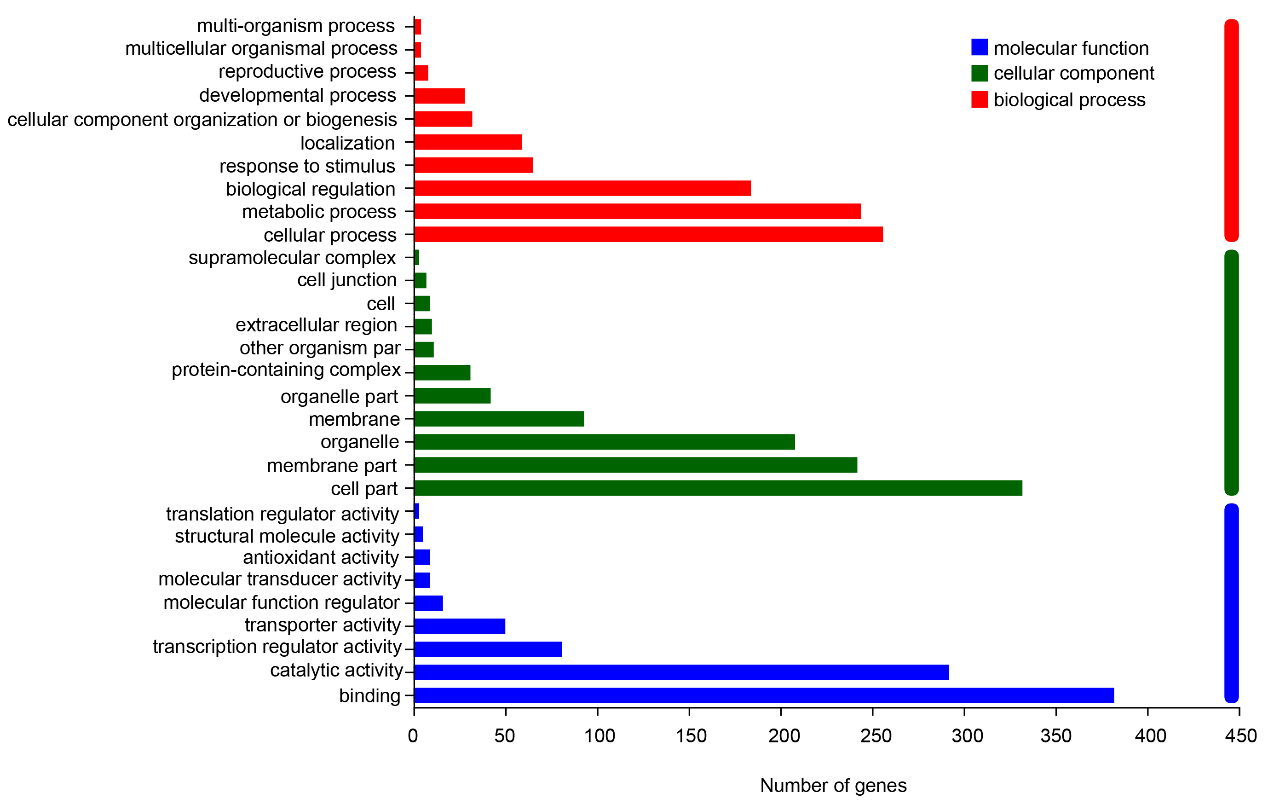


Fig. S2. GO annotation results of CON-5 group and TC50-5 group, focuses on broad categories of gene functions


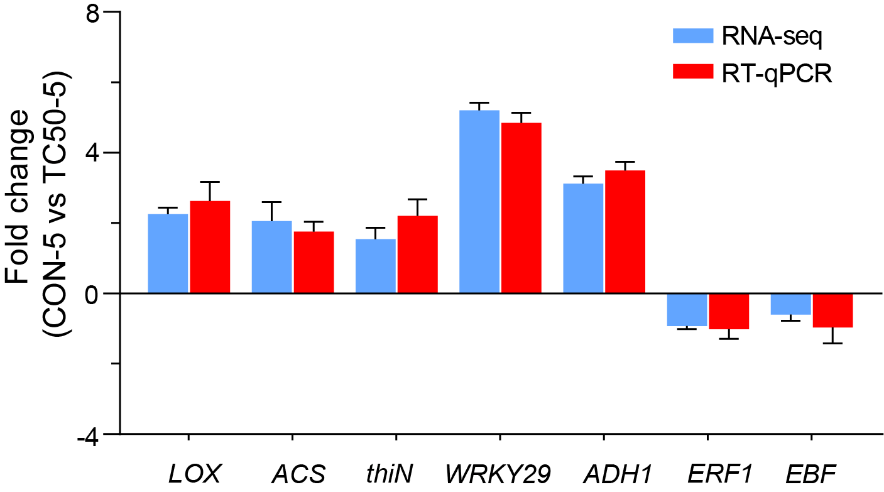


Fig. S3. Validation of transcriptome sequencing results by RT-qPCR


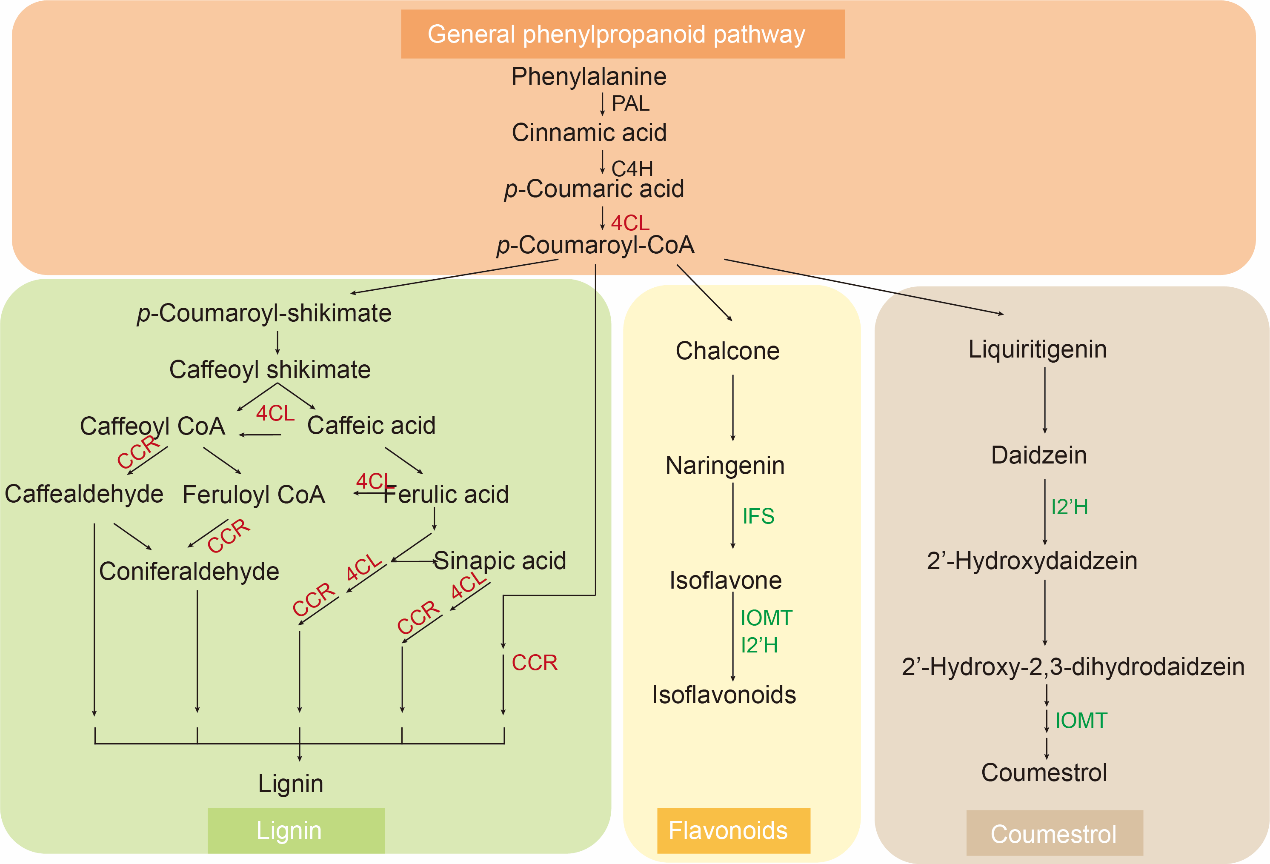


Fig. S4. Effects of tetracycline residues on the synthesis of flavonoids and coumestrol of soybean sprouts during growth. 4CL, 4-coumarate-CoA ligase; CCR, cinnamate-CoA reductase; IFS, Isoflavone synthase. I2'H, Isoflavone 2'-hydroxylase. IOMT, Isoflavone o-methyltransferase.

Table S1 Sequencing data quality control for RNA-seq

| Sample | Clean reads | Clean bases | Error rate (%) | Q20(%) | Q30(%) | GC content (%) |
| --- | --- | --- | --- | --- | --- | --- |
| CON-5-1 | 56022256 | 8101657802 | 0.0245 | 98.23 | 94.77 | 46.02 |
| CON-5-2 | 60874308 | 8769987935 | 0.025 | 98.05 | 94.32 | 45.91 |
| CON-5-3 | 48113816 | 6999704080 | 0.0249 | 98.13 | 94.33 | 46.15 |
| TC50-5-1 | 63099860 | 9095065095 | 0.0243 | 98.31 | 95.05 | 46.07 |
| TC50-5-2 | 49873838 | 7241437012 | 0.0241 | 98.38 | 95.22 | 45.91 |
| TC50-5-3 | 58458968 | 8421230182 | 0.0242 | 98.35 | 95.08 | 45.29 |

Table S2. Tetracycline residues in different organs of soybean sprouts

| Organs | CON | TC25 | TC50 |
| --- | --- | --- | --- |
| Cotyledon | 0.0 ± 0.0^a^ | 0.2 ± 0.0^b^ | 0.4 ± 0.0^b^ |
| Hypocotyl | 0.0 ± 0.0^a^ | 2.9 ± 0.2^b^ | 5.0 ± 1.0^c^ |
| Radicle | 0.7 ± 0.5^a^ | 21.3 ± 4.2^b^ | 37.0 ± 4.2^c^ |

Note: Different letters indicate significant differences from the CON group (*P* < 0.05)
